# Assessment of Resistance of *Varroa destructor* to Formic and Lactic Acid Treatment—A Systematic Review

**DOI:** 10.3390/vetsci12020144

**Published:** 2025-02-08

**Authors:** Yvonne Kosch, Christoph Mülling, Ilka U. Emmerich

**Affiliations:** 1 Institute of Veterinary Anatomy, Histology and Embryology, Faculty of Veterinary Medicine, Leipzig University, An den Tierkliniken 43, 04103 Leipzig, Germany; 2 Institute of Pharmacology, Pharmacy and Toxicology, Faculty of Veterinary Medicine, Leipzig University, An den Tierkliniken 15, 04103 Leipzig, Germany

**Keywords:** *Varroa destructor*, *Apis mellifera*, resistance, formic acid, lactic acid

## Abstract

*Varroa destructor* represents one of the most significant pathogens affecting *Apis mellifera*. A multitude of treatment modalities exist, including those based on pharmaceutical and biotechnological principles. A few years after the first use of synthetic acaricides, *Varroa destructor* developed resistance to these substances, becoming a significant concern. The present review assesses the potential risk of resistance development to formic and lactic acid. This is performed by evaluating sources in the literature over the past 30 to 40 years following the PRISMA 2020 guidelines. Median annual efficacies are determined and evaluated over time. Independent of pharmaceutical form, mite populations are considered non-resistant for efficacies higher than 70%. After the assessment of outliers for lactic acid, the remaining limited data are insufficient to draw conclusions on resistance. The reported unusually low efficacy values for formic acid can be explained by inadequate evaporation rates and study settings. Notably, for both acids, the findings are limited by a low number of efficacy values, and further, more detailed and standardized studies are needed.

## Simple Summary

*Varroa mites* (*Varroa destructor*) are a major pest of honey bees (*Apis mellifera* L.). Untreated colonies usually die within a few years. Therefore, they must be treated with drugs or biotechnological methods. *Varroa destructor* became resistant to synthetic drugs within a few years after their first use, which is a substantial problem. This review explores the risk of resistance to formic and lactic acid by looking at the past 30 to 40 years of research. The median annual efficacy is calculated and evaluated over time, considering values of less than 70% a sign of resistance development. There are few data on lactic acid, so a resistance examination is not possible. The reported unusually low efficacy values for formic acid are caused by evaporation rates and study settings, leaving no indication of resistance development. However, the findings are based on only a few efficacy values, so more studies are needed.

## 1. Introduction

*Varroa destructor* is widespread in bee colonies around the world, causing significant economic damage. After reports from Hong Kong in 1962, this very serious parasite of *Apis mellifera* was first reported in infected German honey bee colonies in 1977, followed by the infection of colonies in the USA in 1987 [1,2,3]. Australia managed to maintain its status as the only continent with honey beekeeping free of the Varroa mite for a long time, but had to abandon eradication programs in 2023 and now also needs other effective control strategies [4]. A large portfolio of control methods has been established, including pharmacotherapeutic treatments and biotechnological methods. The pharmacotherapeutics comprise organic acids, including oxalic, formic, and lactic acid, as well as synthetic acaricides. It has been postulated that organic acids act as contact poisons, and the effect of the acidic pH value is thought to be significant in this context [5,6,7]. Synthetic miticides comprise a range of compounds, including pyrethroids, such as tau-fluvalinate and flumethrin, which act on the voltage-gated sodium channel; the thiophosphoric acid ester coumaphos, an acetylcholinesterase inhibitor; and the amidine amitraz, an octopamine receptor agonist [8]. However, for beekeepers and veterinarians, resistance is an enormous challenge. Resistance is characterized by a reduced or missing medication efficacy of the substance in an initially susceptible population [9]. Although tau-fluvalinate and, in particular, amitraz had an initial efficacy of 90%, the Varroa mite became resistant about 30 years ago (reviewed in [8]). However, amitraz is still the most widely used acaricide against *V. destructor* [10], and reports from some countries, such as Spain, France, and Poland, still show efficacy against *V. destructor* [11,12]. Furthermore, resistance to flumethrin and coumaphos has emerged and evolved over time [8]. Resistance to synthetic acaricides is suggested to be caused by target structure insensitivity and metabolic resistance [8,13].

With the *Guideline on veterinary medicinal products controlling Varroa destructor parasitosis in bees*, the European Medicines Agency (EMA) has established a comprehensive framework for evaluating the reliability of varroacides [14]. The efficacy of the medicinal products is influenced by various factors, including the mode of application, the quantity and frequency of treatments, the dosage of the product, the status of the brood in the hives, and climatic conditions [14]. The observed variability in the efficacy of amitraz against *V. destructor*, as described by the EMA, is thought in part to be due to similar factors [15]. A single mutation in the target structure does not always lead to a decrease in efficacy [12,16].

Given these developments, alternative, more effective control methods are even more important. The use of oxalic, lactic, and formic acid—which are commonly used alone and in combination with synthetic acaricides—has increased [10]. In the USA, *V. destructor* is often treated with formic acid as the sole varroacide or in combination with other substances [17]. In Germany, these three acaricidal organic acids have been approved for use and are freely available for treating Varroa mites [18].

Although formic acid is thought to negatively impact honey bee welfare and hive growth [19,20,21,22], depending on the level of Varroa infestation, slowly released formic acid may support hive development by effectively controlling *V. destructor* [20]. Formic acid is the only approved organic acid capable of controlling not only the mites that infest adult bees but also the mites that are present in capped brood [18]. The mode of action of formic acid is not fully understood. However, the binding of cytochrome c oxidase has been demonstrated to inhibit the respiratory chain, which is known to lead to oxidative stress and acidosis with subsequent cell death [5]. Next to oxalic acid, formic acid is the most widely used organic acid in Europe [10]. Formic acid is a naturally occurring compound in honey and related products. However, the maximum permitted level of free acids in honey is 50 milliequivalents per kilogram [23]. Formic acid-resistant Varroa mites are not currently known (reviewed in [19]). Given the accelerated development of resistance to synthetic varroacides, possibly as a consequence of their overuse, it is essential to investigate the possibility of developing resistance to such acids [3].

The following review aims to evaluate whether *V. destructor* has developed resistance to treatment with lactic and formic acids based on published reports of acaricidal efficiency in recent decades. Oxalic acid was previously addressed in [3].

## 2. Materials and Methods

This systematic review of the literature pertaining to lactic and formic acid treatment was conducted according to the PRISMA 2020 guidelines [24]. As reported in [3] for oxalic acid, original peer-reviewed articles were identified from a search of databases (PubMed^®^ and Web of Science^TM^) and the catalogs of the German National Library and the Library of the University of Leipzig.

**Figure 1:**
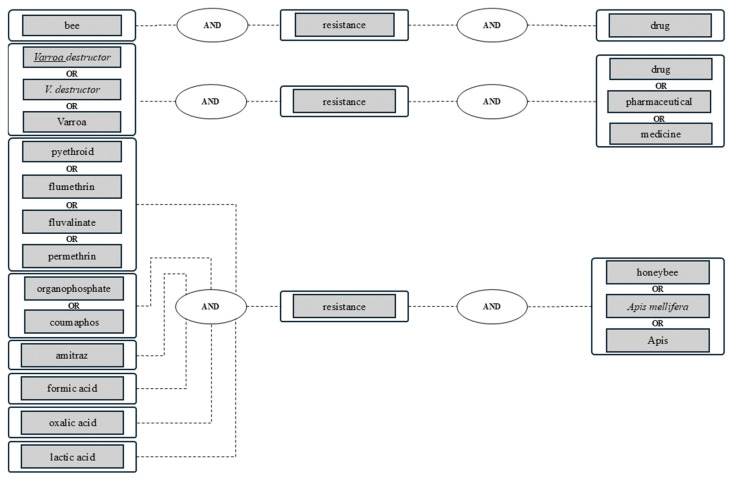
Keywords and examples of logical combinations. Interval of search and alert: 1 January 2023 to 31 December 2023. Searched databases: PubMed^®^, Web of Science^TM^, German National Library, and Library of the University of Leipzig [3].

For comprehensive results, several synonym combinations were selected for the full-text search (Figure 1) [3].

All articles identified by the search were analyzed using the same methodology described in [3]. In brief, this comprised (1) screening and selecting all articles whose abstracts met the inclusion criteria, such as treatment methods against *V. destructor*, including the treatment success achieved, resistance mechanisms, or adverse effects of organic acids on *Apis mellifera*; (2) removing duplicates; (3) reviewing the full texts of the remaining publications for essential information on the efficacy of formic acid and lactic acid against *V. destructor*; and (4) systematically documenting the parameters relevant for the evaluation and discussion.

Deviating from the PRISMA 2020 recommendations, the selected publications were screened by only one person instead of at least three reviewers. This study aims to examine efficacy development trends; the potential for bias is therefore not a significant concern, as any potential bias resulting from the subjective view of a single reviewer would equally impact efficacy values over time, leaving trends unaffected [3]. As in [3], maps of the study locations were created utilizing the Quantum geographic information system (Qgis) [25].

### Data Processing

To make published doses comparable, they were standardized with respect to hive volume, as shown in Equation (1). This volume dose was chosen because most of the time, “fumigation” was chosen as the application method. The original unit was sometimes retained due to missing information for calculating the volume dose according to Equation (1).

For most years, there have only been a few reported efficacy values, if any. In addition, those values have frequently been widely spread. Therefore, the annual median was used for the analysis. The efficacy values were grouped according to the organic acid used and the pharmaceutical form.

**Equation (1):** Calculation method for formic and lactic acid, where $D_{V}$: volume dose of hive volume as target dose; $n_{T}$: number of treatments; $n_{M}$: number of used carrier matrices or dispensers; *c*: concentration (mass/volume); *v*: volume of formic or lactic acid per treatment; l: hive length; w: hive width; and h: hive height.

(1) $$D_{V}=\frac{n_{T}\times n_{M}\times c\times v}{l\times w\times h}$$

The normalized doses were grouped by pharmaceutical form. An analysis of dose changes over the years was facilitated by calculating an average annual dose across all pharmaceutical forms. The average rather than median value was chosen, as the doses were not as widely scattered as the efficacy values and the number of extremely high and low values was limited.

## 3. Results

**Figure 2:**
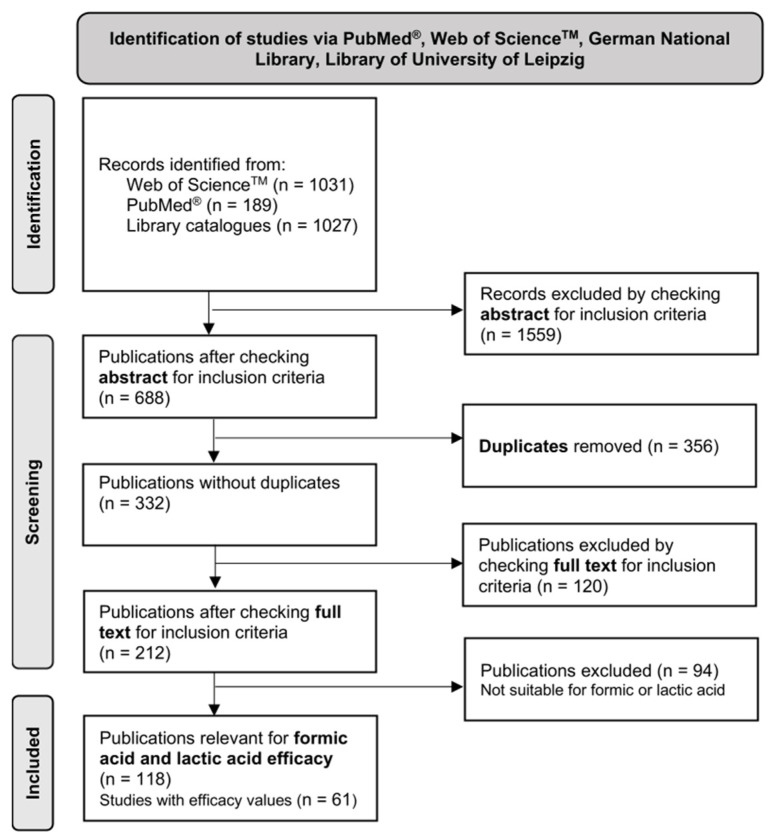
Procedure for a PRISMA 2020 guidelines-compliant literature search [24].

An initial set of 2247 references, which was also the starting point in [3], originated from different sources, as shown in Figure 2. Of these, the abstracts of 688 sources met the inclusion criteria. However, due to duplicates, they only corresponded to 332 distinct publications [3]. In a subsequent comprehensive textual review, 212 sources were identified as pertinent to the study’s objective, with 118 deemed relevant to the observations on treating *V. destructor* with formic or lactic acid. While certain sources lacked efficacy data, they contained pertinent facts about the efficacy assessment of formic or lactic acid treatments against V. destructor in general. This information relates, for example, to the seasonal mite infestation level and path of infection [26], the possible mode of action of formic or lactic acid [27,28,29,30,31], formic or lactic acid residues in bee products [32], and the impact of ambient temperature and weather conditions on treatments with formic or lactic acid [33,34,35]. The “References” Section consists only of sources that contain individual efficacy values or essential aspects for the discussion.

### 3.1. Efficacy Measurement

Like in the oxalic acid treatment studies reviewed in [3], mite counting and efficacy evaluation techniques varied across the formic and lactic acid treatment studies. Washing, bottom board sampling, and brood cell opening were the three main methods of mite counting, practiced individually or in different combinations. The number of counts for an individual treatment varied from 2 [36] to 30 [37], with frequencies of 1 [37,38] to 14 [39] days. The “washing method” refers to harvesting approximately 200 to 300 bees and detaching the mites by rolling [40,41,42] or shaking [43,44,45,46,47] them in benzine [45], ether [40,41,42,46], alcohol [47,48,49], icing sugar [50], or water with and without detergent [43,51,52]. After this, the mites are quantified to estimate the infestation level. “Bottom board sampling”, the most common method for determining efficacy, is performed by counting mites that have fallen from the colony on sticky bottom boards [45,53,54,55,56,57,58,59]. Mites in 200 to 300 opened brood cells per hive were counted in some trials [36,60,61,62,63,64] to determine infestation levels. In several reports, the brood was described as either drone or worker brood but remained uncharacterized in others.

The efficacy values were calculated in different ways. However, sometimes, details regarding the calculation method were not described. The majority of studies used one of the methods described in [3].

### 3.2. Formic Acid

**Figure 3:**
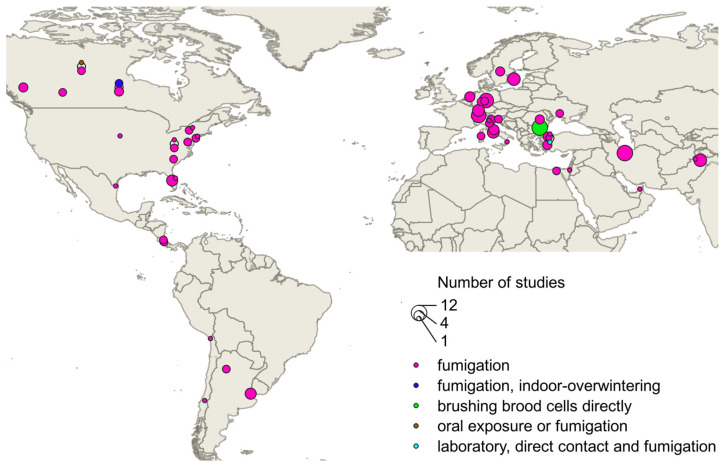
Geographical distribution of formic acid studies that could be assigned at least at the country level. The size of the circle indicates the number of studies with the same localization.

A total of 161 individual efficacy values resulting from treatment with formic acid were found in 55 sources. The values resulted from field trials (n = 140), laboratory trials (n = 9), or reviews and theoretical treatment concepts (n = 12), using fumigation, fumigation combined with overwintering indoors, brushing brood cells directly, and oral and direct exposure as application methods. The geographic distribution of these studies is shown in Figure 3.

**Figure 4:**
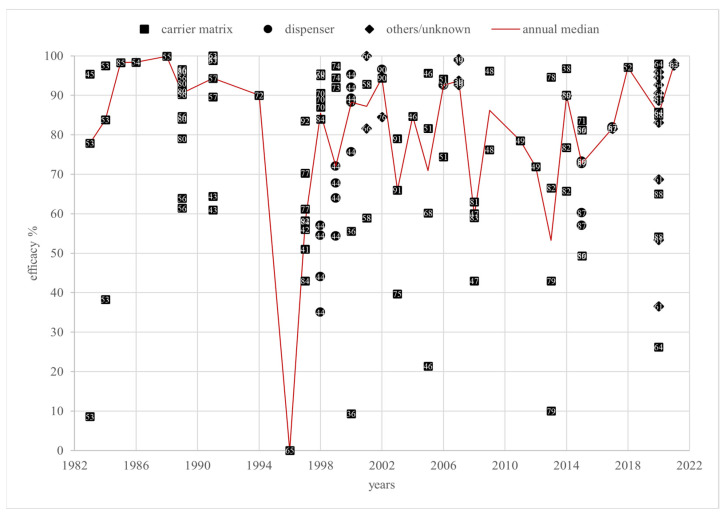
Efficacy values depending on the pharmaceutical form and annual median efficacy of treatment with formic acid under field conditions. References [36,37,38,39,41,42,43,44,45,46,47,48,49,50,51,52,53,54,55,56,57,58,59,60,61,62,63,64,65,66,67,68,69,70,71,72,73,74,75,76,77,78,79,80,81,82,83,84,85,86,87,88,89,90,91,92] correspond to the numbered data points.

Field trials are the most relevant for practical use and are, therefore, the only ones discussed in this review. The 140 individual efficacy values of the field trials varied from 0% [65] to 100% [63,66] between 1983 [45,53] and 2021 [62]. These values were reported by only fifty-five sources, as some of them included more than one trial. A total of 94 (67.1%) and 54 (38.6%) efficacy values exceed 70% and 90%, respectively. Out of 32 annual medians over a 38-year period, 20 years were below 90%, but only 5 years were below 70% efficacy (Figure 4). The efficacy values were measured in 25 countries. The efficacy values originate from 9 broodless colonies and 77 colonies with brood. The remaining 54 had an unknown brood status.

The treatments can be divided into three groups depending on the pharmaceutical form of formic acid (Table 1): carrier matrix (n = 95), dispenser (n = 22), and others/unknown (n = 23). In the “carrier matrix” group, the hive air is fumigated with formic acid from a base material. Depending on the study setting, different materials were used: cardboard [36,46,92], pressed wood [45], oasis sponge [81], Dri-Loc^®^ pads [49], newspaper sheets [65], unspecified or tree fiber material [42,56,63,68], and cotton [54,83]. The “Illertisser Milbenplatte” [53,56,70,80,85] and “West Virginia formic acid fumigation board” [47] were used several times. Gel-based carrier materials like MAQS^TM^ [38,48,86], “Beltsville Formic Acid gel package (BFA)” [77], “BeeVar” gel packets [73], a formic acid-gel package called a “formic acid dispenser” in [74], and the Varterminator^®^ [78,86] were also used. Some of these carrier matrices were placed in a porous plastic bag [55,58,71,75,79] or a “non-woven fabric” [78,86]. The pharmaceutical form “dispenser” characterizes the fumigation of liquid formic acid using a device that ensures the slow and regulated release of the active substance. Devices like a wick applicator [44], the Nassenheider Professional^®^ [59,67,86,87], the MHT^®^ Universal-Evaporator with a regulable opening size [37], the Liebig Dispenser [90], and the Aspro-Nova-Form^®^ Dispenser [67] were used. The “others/unknown” category includes studies that did not specify the fumigation method [40,50,52,60,66,76] and those that did not fumigate but brushed the formic acid solution directly on brood cells and put the frames back into the hive [61,62,64].

The doses were calculated using Equation (1). Soaking two pads, each with 100 mL of formic acid 60%, and placing both in a Dadant Blatt hive, a volume dose of approximately would be administered to the colony.

$$\frac{1\times 2\times 0.60\times 100\;mL}{4.35\;dm\times 4.35\;dm\times 3.33\;dm}\approx 1458\;mg/{dm}^{3}$$

Using Equation (1), the recommended maximum and minimum doses for long-term and short-term treatment according to [5] can be determined. For a long-term treatment of 10 days, a dose of 8000 to 12,000 milligrams of formic acid per 40 dm^3^ per day is advised [5]. Consequently, a daily volume dose of 200 to 300 mg/dm and an overall volume dose of 2000 to 3000 mg/dm should be administered. For a short-term treatment with a hive volume of 60 dm, 40 milliliters of 60% formic acid are described in [5]. Therefore, 24,000 milligrams per hive and treatment or 400 mg/dm per treatment should be used.

**Figure 5:**
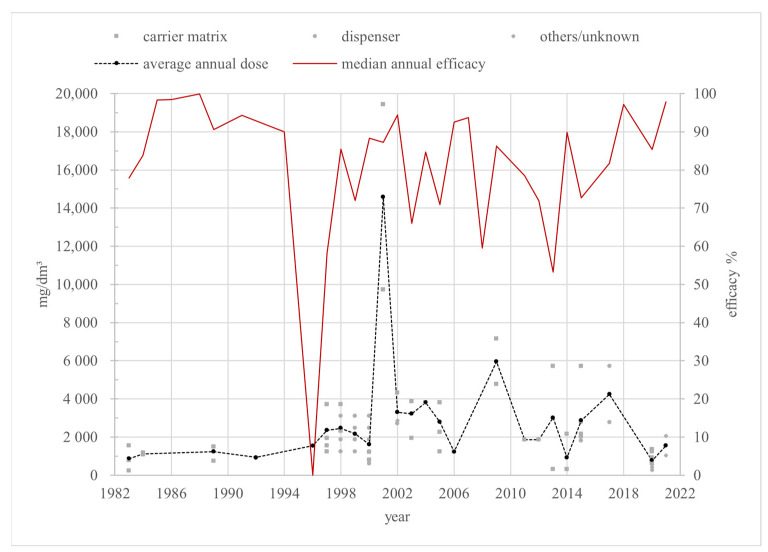
Total doses and annual average doses of formic acid.

The amount of formic acid solution used varies from 9.1 [45] to 300 milliliters per carrier matrix or dispenser [48,68], with a concentration of 36% [78] to 98% [45]. In some sources, the hive type and volume [39,43,47,50,52,53,54,56,59,60,63,70,72,73,79,81,83,85,92], amount, or concentration of formic acid [55,57,64,66,80,89] are not noted, and the volume dose is not calculable. The dose modifications over time and the annual average doses are shown in Figure 5.

The annual average dosages over all three pharmaceutical forms range from 758 mg/dm in 2020 to 14,571 mg/dm in 2001. All reported doses in 2001, with the pronounced maximum, belong to the “carrier matrix” category. Figure 5 shows two more peaks of annual average doses after 2001: in 2009 with 5952 mg/dm and in 2017 with 4238 mg/dm. Only two doses can be calculated for 2009, both from the “carrier matrix” group using Mite-Away Quick Strips^TM^. The annual average dose in 2017 consists of two doses from the “dispenser” category. In most years, the average dose remains below 4000 mg/dm; 70.8% of the annual average doses fall between 400 mg/dm and 3000 mg/dm.

### 3.3. Lactic Acid

**Figure 6:**
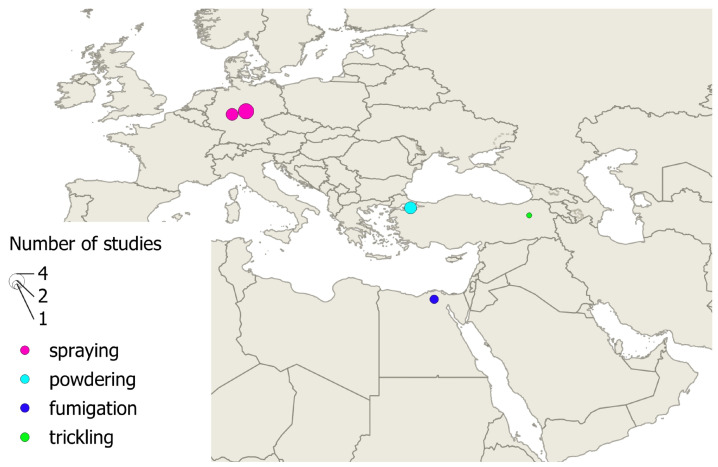
Geographical distribution of lactic acid studies. The size of the circle indicates the number of studies with the same localization.

Only thirteen individual efficacy values of lactic acid were found in six literature sources. Some reported more than one trial setting. All were conducted as field trials. Spraying (n = 7) [93,94,95], fumigation (n = 2) [43], powdering (n = 3) [46], and trickling (n = 1) [96] were used as application methods. The studies took place in only four countries, as shown in Figure 6.

**Figure 7:**
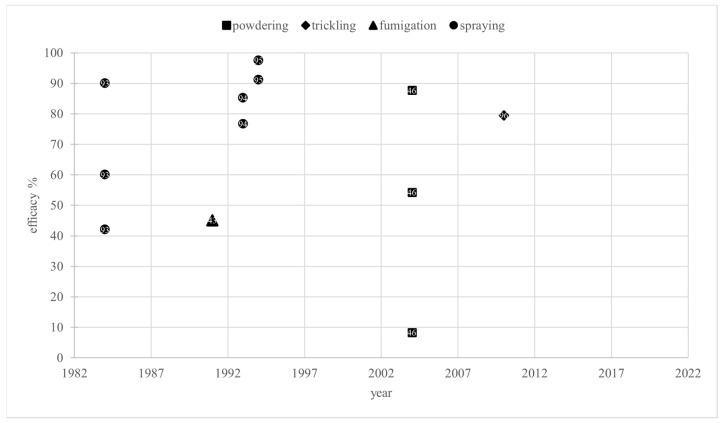
Efficacy values of treatment with lactic acid over time depending on application method. References [43,46,93,94,95,96] correspond to the numbered data points.

The efficacy values ranged from 8.3% [46] to 97.5% [95]. They were reported in studies from 1984 to 2010. Due to the limited number of data points, an annual median efficacy was not calculated (Figure 7).

The only calculable dose is 53.6 mg/dm. It is impossible to calculate the remaining doses due to a lack of information on the type and volume of the hive. A volume of five [46,93,94,96] to eight [95] milliliters of lactic acid with a concentration of 15% [46,93,95,97] to 40% [43,94] was applied.

Due to the small amount of data, lactic acid is not discussed further in this review.

## 4. Discussion

This systematic review aims to evaluate whether *V. destructor* has developed resistance to treatment with lactic and formic acids based on reports of acaricidal efficiency in recent decades.

As described in [3], the European Medicines Agency (EMA) defines the efficacy of a varroacide as the percentage of mite mortality, calculated by dividing the number of fallen mites after the tested treatment by the total number of fallen mites after the tested treatment and a critical test, multiplied by 100 [14]. Almost all efficacy values shown in Figure 4 and Figure 7 were determined this way. According to the EMA’s *Guideline on veterinary medicinal products controlling Varroa destructor parasitosis in bees* [14], an efficacy of more than 90% for non-synthetic varroacides should be achieved to reduce the risk of resistance emergence. To determine efficacy, a standardized test protocol should be used based on the following criteria: fallen mites should be counted by using the bottom board sampling method pre- and post-treatment, and the test treatment should be followed by a treatment with a chemically unrelated substance with a documented efficacy of more than 95%, called a “critical test” [14]. In line with the EMA’s emphasis on the pharmaceutical control of varroosis being only one part of an integrated pest management (IPM) system, organic acid treatments should be combined with other control methods to ensure an overall efficacy of at least 90%. This review only considers formic acid or lactic acid as the sole agent in a number of studies. In line with the approach taken for oxalic acid in [3], in this review, an efficacy of at least 70% for treatment with these organic acids, without any additional substances or control methods, is considered adequate, effective, and not indicative of resistance, as the combination of several such equally effective controls will ensure sufficient overall efficacy.

Aside from efficacy, changes in dosage over time may also indicate the emergence of resistance. Should the dosage increase while the efficacy remains constant, it would be possible that the resistance of the target is compensated by a higher dosage [3]. Consequently, it is necessary to rule out the possibility of resistance being masked by dose escalation.

In this study, the efficacy of formic acid over the years was evaluated to investigate the initial question of resistance development. In accordance with EMA recommendations for efficacy studies, the pharmaceutical form, dose, quantity of medications, and experimental setting were analyzed. The status of the brood is of less importance given that formic acid is effective at reaching mites even within capped brood cells [36,47,60,63,64,70,89,98]. In addition, two other factors were examined. Firstly, the impact of escalating doses over time in conjunction with maintaining a consistent level of efficacy was analyzed. Secondly, our investigation focused on dose adjustments immediately following lower efficacy levels.

Treatment with lactic acid was not analyzed due to insufficient data.

### 4.1. Efficacy Evaluation

In most cases, the reported efficacy values of formic acid were calculated by dividing the number of fallen mites after treatment by the total number of fallen mites, including those killed by the critical test.

Bottom board sampling, as recommended by the EMA for mite counting [14], contributed 64% of the formic acid efficacy values. The EMA’s new *Concept paper on the revision of the guideline on veterinary medicinal products controlling Varroa destructor parasitosis in bees*, published in July 2024, also supports the idea of counting mites using the washing method [99]. Combined, those two counting methods cover 73% of the reported efficacy values. As the remaining studies use a very wide range of efficacy determination methods, this aspect will not be discussed in detail but will be considered for efficacy outliers where appropriate. The differences in mite counting methods limit the comparability of the results, highlighting the need for standardized testing.

### 4.2. Formic Acid 

In 38 years of formic acid field trials from 1983 to 2021, there were only 5 years with an annual efficacy median below 70%, while 11 years showed an annual median efficacy exceeding the EMA-recommended 90% solely with formic acid treatments. In particular, the last reported year, 2021, achieved an annual median efficacy of 97.8%, strongly suggesting the sustained efficacy of formic acid treatments against *V. destructor*.

The median efficacy in 1996 was 0%, derived from a single study with an efficacy value of 0% conducted in Lincoln, Nebraska [65]. Brood-positive “two-deep” Langstroth hives were treated through one-time fumigation over 45 days out of newspaper sheets in a porous plastic bag, initially placed above for 24 h and, for the remaining time, below the brood chamber [65]. In total, 200 milliliters of a 65% formic acid solution was used [65], resulting in a total volume dose of 1548 mg/dm over 45 days. The approved total dose of formic acid for a long-term treatment over 10 days ranges between 2000 and 3000 mg/dm or between 200 mg/dm^3^ and 300 mg/dm^3^ per day [5]. The total dose in [65] was about half of the recommended one. Spreading it over potentially 45 days, the daily dose could have been as low as 20 % of the recommended value. Therefore, the administered dose was too low, potentially by a wide margin, for an effective treatment. In addition, except for the first 24h, the carrier matrix was placed below the chamber, but formic acid has a higher density than air, preventing it from ascending into the hive. Thus, this placement severely limited the amount of active substance reaching the mites. Therefore, the observed inefficiency was a consequence of an insufficient dose and inadequate application and provides no indication of resistance development.

The next notable year is 1997, with an annual median efficacy of 58.1%, deriving from five different studies with values of 51% [41], 56% [42], 70.3% and 61.2% [77], 58% and 43% [84], and 58.2% and 83.5% [92]. All used a carrier matrix. The results reported in [92] were obtained from studies in Los Santos, Costa Rica, via bottom board sampling and the washing method, using Apistan^®^ (tau-fluvalinate) strips for the critical test. Three applications using 15 mL of an 85% formic acid solution soaked in a piece of cardboard seven days apart, placed below the hive, resulted in 83.5% efficacy, while three applications of 10 mL resulted in only 58.2% efficacy. The steep drop in efficacy suggests marginal to insufficient doses. The *summary of product characteristics* for formic acid, published by Serumwerk Bernburg AG, recommends a dose of 24,000 mg per chamber for each short-term treatment, with the carrier matrix placed above a 60 dm hive [5]. The hive volume was not specified in [92], but the individual doses of 12,750 mg and 8500 mg of formic acid are significantly lower than the recommendation. In addition, the carrier matrices in [92] were unsuitably placed under the chamber, reducing the amount of acid reaching the mites even further, as explained above. Therefore, the low efficacy of 58.2% cannot be attributed to resistance development but to the low dosage and inappropriate placement of the carrier matrix.

The trial in [41] achieved an efficacy value of 51% by placing fiber material soaked with 250 milliliters of 65% formic acid in a porous plastic bag above a “two-deep” Langstroth hive for 33 days, quantifying mites via washing and bottom board sampling and using Apistan^®^ in the critical test [41]. However, a low temperature of 4.8 °C to 16.8 °C—instead of 12 °C to 30 °C, as recommended in [5]—resulted in only 104 to 114 g of fumigated 65% formic acid solution, reported by the authors of [41]. Consequently, the actual total volume doses amounted to 805 mg/dm to 885 mg/dm or average daily volume doses of 24.4 mg/dm^3^ to 26.8 mg/dm^3^, failing short of at least 2000 mg/dm or 200 mg/dm^3^/d, respectively, for long-term treatment [5], by a wide margin. Thus, the observed low efficacy was very likely not caused by the development of resistance but rather by the low temperature and associated low evaporation rate. The same conclusion can be drawn from the study described in [42], which obtained an efficacy value of 56% in average temperatures ranging from 4.06 °C to 19.01 °C using the same study setting as in [41]. A total evaporation of only 158 g of 65% formic acid or an average rate of 5.26 g per day was achieved. The resulting actual total and average daily volume doses were 1220 mg/dm and 37.0 mg/dm^3^/d, respectively, which, again, are significantly lower than the recommended doses.

The proposition of insufficient evaporation is supported by a similar experiment: a total actual evaporation rate of 328 g of 65% formic acid resulted in an efficacy of 95% [68]. The authors did not specify a daily dose, and a calculation of the exact daily dose is not possible, as the release of formic acid depends on a number of factors, like temperature and humidity, and, therefore, occurs irregularly. The calculated total volume dose, however, was 2540 mg/dm, within the recommended range of 2000 mg/dm to 3000 mg/dm.

A study by Feldlaufer et al. achieved efficacy rates of 61.2% and 70.3% with two different treatment regimens [77]: one administered four equal doses of formic acid using an absorbent pad, and the other, a single “Beltsville Formic Acid” gel package (BFA). Both were tested in “two-deep” Langstroth hives [77], monitoring formic acid levels in the hive air. The four short-term treatments used absorbent pad-administered volume doses of 309.5 mg/dm and 1238 mg/dm in total, lower than the recommended 400 mg/dm for short-term treatment and 2000 mg/dm for long-term treatment.

Moreover, the absorbent pads rapidly dried out, letting hive air concentrations drop below 10 ppm on multiple occasions [77], leading to a low efficacy of 61.2%. The long-term treatment using the BFA with a volume dose of 1548 mg/dm, still below the recommendation, maintained a stabler and higher formic acid concentration of never less than 10 ppm and up to 50 ppm, leading to an efficacy of 70.3% [77]. Thus, the low efficacy of the absorbent pad is a consequence of the low dosage and inadequate release rather than an indication of efficacy loss.

The remaining values from 1997 came from one study. Melathopoulos et al. reported three different efficacy values: 58% and 43% from 1997 and around 84% from 1998 [84]. All resulted from six short-term treatments, using absorbent pads as the carrier matrix soaked with 40 milliliters of 65% formic acid [84]. The total volume dose for all treatments was 3714 mg/dm, with partial doses of 619 mg/dm. The critical test was performed using Apistan^®^ strips [84]. The sole differentiating factor between the achieved efficacies was the ambient temperature. The efficacies were found to be 58% at 18 to 35 °C, 43% at 8 to 15 °C, and 84% at 12 to 25 °C [84]. The recommended ambient temperature range for formic acid treatment is 12 to 30 °C [5]. Only the treatment in the recommended temperature range resulted in an efficacy higher than 70%. This leads to the assumption that the temperature and, consequently, the evaporation rate were the reason for the lower efficacy and not the development of resistance.

The 66% annual median in 2003 originates from three studies with efficacies of 39.7% [75], 66% [91], and 79% [91]. All used a plastic-covered soaked pad as a carrier matrix for long-term treatment in Langstroth hives, 42 days in [75] and 30 days for both studies reported in [91].

The mites in [75] were counted via alcohol washing before and after the treatment and were resistant to fluvalinate and coumaphos [75]. A critical test was not performed [75]. The total dose was 3869 mg/dm. The evaporation rate was determined after each week, starting with 98.1 g of 65% formic acid, equivalent to a daily volume dose of 217 mg/dm in week one, dropping to 78.2 g or 173 mg/dm in week 2, and ultimately decreasing further to 42.2 g in the fourth week or a daily dose of 92.9 mg/dm. While the rate may just have achieved the recommended minimum of 200 mg/dm per day for 10 days, an average daily rate of around 90 mg/dm per day in weeks 3 and 4 is too low for sufficient mite control. To compare the post-treatment infestation rate with the pre-treatment rate, the mites were counted weekly via the alcohol washing method before the initial treatment and for 6 weeks afterward [75]. Therefore, the mites were counted for four weeks longer than the recommended acid concentration was maintained, potentially enabling the re-infestation and reproductive growth of the mite population.

Thus, the post-treatment population of mites on the adult bees may be overestimated, masking the initially positive effect of the formic acid. The lack of a critical test and inadequate dosing for the period of mite monitoring indicate that the low efficacy value of 39.7% should not be overestimated.

Stanghellini et al. monitored mites via alcohol washing and bottom board sampling [91]. An administered dose of 163 g resulted in an average evaporation of 4.82 g per day and a total evaporation of 145 g. The total volume dose of formic acid actually evaporated was, therefore, 1120 mg/dm overall, or 37.3 mg/dm per day, well below the recommended 2000 mg/dm and 200 mg/dm/d, respectively, thus fully explaining the low efficacy of 66%. The low median efficacy of 2007, therefore, does not indicate resistance development.

All reported efficacy values reported in 2008 were less than 70% and belong to the carrier matrix category. The specific hive type or volume used in the studies was not reported. Mahmood et al. used 13 g of formic acid by administering 20 g of 65% formic acid in a short-term treatment repeated three times [83] instead of the recommended 24 g of formic acid for each short-term treatment [5]. In addition, the carrier matrix was—ineffectively, as explained above—placed below the hive. Thus, the resulting efficacy of 59% must be attributed to insufficient acid levels in the hive air. A four-time treatment with 12 g of formic acid, also only half of the approved dose, achieved 63% efficacy in [81]. The low efficacies in [81,83] were clearly the consequences of low doses and carrier matrix misplacement. A single short-term treatment with 37.5 g of formic acid for 17 h described in [47] reduced mite infestation by 43% and 60%. Considering that four or five repetitions for a short-term treatment are recommended in [5], a single treatment cannot be expected to be sufficiently effective. Overall, the annual median efficacy value of 59.5% in 2008 was caused by insufficient doses or inadequate treatment and does not indicate the development of resistance.

The annual median efficacy in 2013 was 53.2%, derived from three studies with efficacy values of around 10% and 40% in [79], 66.5% in [82], and 94.6% in [78]. The authors of both [79] and [82] applied formic acid with a carrier matrix placed below the hive. The hive type or volume in [79] was not reported. An efficacy of around 10% was achieved through two short-term treatments, each with 12.8 g of formic acid [79], about half of the 24 g recommended in [5]. The administered dose and application position below the chamber resulted in markedly reduced efficacy. A long-term treatment for 40 days with a daily evaporation of 8 to 12 g of an 85% formic acid solution in the same publication led to an efficacy of around 40% [79]. The hive size or type was not reported. A reported evaporation minimum of 8 g and a maximum of 12 g of 85% formic acid per day resulted in a minimum daily evaporation of 6800 mg of formic acid and a maximum daily evaporation of 10,200 mg per chamber. An amount equal to 8000 to 12,000 mg of formic acid per chamber is recommended for a long-term treatment [5]. Therefore, the administered dose of 6800 mg is below the recommended minimum of 8000 mg of formic acid. A calculated maximum daily evaporation per chamber of 10,200 mg was exactly in the dose range but still below the recommended maximum of 12,000 mg. Therefore, it can be surmised that in most cases, the administered dose, combined with the placement below the chamber, is too low for a sufficient efficacy of more than 70%. The 66.5% efficacy in [82] was reported for a long-term treatment in winter between November and January with “soaked clothes” below the hive. The trial was located in Islamabad, Pakistan. The temperature, humidity, and the exact time period of application were not reported. In total, 20 g of 65% formic acid were placed under a Langstroth hive. A calculated total volume dose of 310 mg/dm, combined with an inadequate carrier matrix position, was far too low for sufficient efficacy compared with a recommended total dose of at least 2000 mg/dm for long-term treatments. Thus, none of the outliers in 2013 suggest resistance development, as all can be traced to insufficient dosages in combination with ineffective carrier matrix placement.

In summary, none of the reported low efficacy levels support a resistance hypothesis. Furthermore, to eliminate the possibility of resistance being hidden by alterations in the formic acid dosage, it is necessary to assess variations in dosage over time, as previously outlined.

When comparing dose and efficacy time series, in most cases, no decrease in the median efficacy can be observed immediately before a formic acid volume dose increase. Only from 2008, with an annual median efficacy of 59.5%, to 2009, with an efficacy of 86.2%, did the average total dose increase from 1238 mg/dm in 2006 to 5952 mg/dm in 2009. The volume dose for 2006 can be calculated as 1238 mg/dm. The study in that year used a four-time short-term treatment, placing a carrier matrix above the hive, with sufficient efficacy outcomes of 94.1% and 74.45% [51]. In 2008, the efficacy decreased to 59.5%. As described above, the low efficacy in 2008 was not due to the development of resistance but to a low dose in general. As there is no information on hive type or volume, a volume dose cannot be determined for 2008. In 2009, the median efficacy increased to 86.2% combined with an increase in total dose to 5952 mg/dm. In contrast to 2006 and 2008, the bee hives were treated via the long-term application of Mite-Away Quick Strips (MAQS^TM^) [48]. The total dose applied with MAQS^TM^ had to be higher, as the formic acid was coated with a gel formulation, resulting in a slow release of the active ingredient over a longer period. The actual dose in the hive air was not reported, making it difficult to compare doses. The volume dose for the approved short-term treatment was only around 1600 to 2000 mg/dm over all treatments compared with a long-term treatment of about 2000 mg/dm to 3000 mg/dm. Therefore, the dose increase was caused by different treatment regimens requiring different dose levels. There is no resistance masked by dose escalation.

Another notable annual average dose was 14,571 mg/dm in 2001. This is the highest annual average dose for the whole period from 1983 to 2021. The dose was related to a sufficient median efficacy level of around 87%. The average dose consisted of one study with different trial settings [58]. The volume dose of formic acid ranged between 9714 mg/dm and 19,429 mg/dm. Formic acid was applied by carrier matrices described as soaked pads under plastic sheets with two holes to create an evaporation chamber [58]. The treatment duration was not reported, but the pharmaceutical form allows for the assumption that a stable long-term application was intended. An evaporation rate was not provided [58]. The aim was to evaluate the efficacy of different carrier matrix placements in subtropical and temperate climates [58]. This intention suggests that the formic acid dose was not the focus of the study; rather, the authors wanted to ensure the highest possible efficacy and did not want to risk masking the sufficient efficacy of different placements with a possibly insufficient dose. Resistance development masked by higher doses seems unlikely, especially as the following year, 2002, utilizing a lower average dose of 3291 mg/dm, showed a higher and sufficient median efficacy of 94.4%, not indicating any resistance development.

Throughout the remainder of the observation period, the average annual dose remained relatively constant, between 400 mg/dm and 3000 mg/dm, which is the recommended dose range for a single treatment in the short-term and long-term concepts, along with a largely consistent efficacy rate of over 70%.

Finally, there was no indication of covert resistance in *V*. *destructor* to formic acid.

## 5. Conclusions

A comprehensive review of the relevant literature reveals a paucity of evidence indicative of *V. destructor* developing resistance to formic acid over the past four decades. Irrespective of the pharmaceutical form, the efficacy is, in the majority of cases, in excess of 70%. Lower efficacy values can be clarified by referring to the divergent study characteristics. The influence of the actual evaporation rate on the efficacy of formic acid treatment emphasizes the need for detailed evaporation monitoring for robust efficacy studies. The ideal way to accomplish this would be to determine the actual formic acid concentration in the hive air over time.

The limited number of efficacy values and their wide range necessitated calculating medians. While these values do not indicate resistance to formic acid, there is currently no robust evidence to completely rule out the possibility of resistance development. Further research and standardized testing are essential, as the divergent circumstances and experimental settings evidently restrict the validity and comparability of the results.

## Figures and Tables

**Table 1:** Pharmaceutical forms of formic acid.

Pharmaceutical Form	Dose Range (mg/dm)	Number and Share of Efficacy Values	Efficacy			References
			Range (%)	Fraction ≥70%	Fraction ≥90%	
Carrier matrix	232–19,429	95 (67.9%)	0–100	61	37	[36,38,41,42,43,45,46,47,48,49,51,52,53,54,55,56,57,58,59,63,64,65,68,69,70,71,72,73,74,75,77,78,79,80,81,82,83,84,85,86,88,89,90,91,92]
Dispenser	607–5714	22 (15.7%)	35–96.5	13	4	[37,39,44,59,67,86,87,90]
Others/ unknown	270–2857	23 (16.4%)	36.5–100	20	13	[40,50,52,60,61,62,64,66,76]

## Data Availability

Newly generated data are described in the article. They were generated from existing data from different literature sources shown in the “References” Section.
